# Two Phenotype-Differentiated *Acinetobacter baumannii* Mutants That Survived in a Meropenem Selection Display Large Differences in Their Transcription Profiles

**DOI:** 10.3389/fmicb.2019.02308

**Published:** 2019-10-09

**Authors:** Qianqian Gao, Xiaobin Meng, Hanfu Gu, Xueqin Chen, Huaqing Yang, Yangyang Qiao, Xuemin Guo

**Affiliations:** ^1^Zhongshan School of Medicine, Sun Yat-sen University, Guangzhou, China; ^2^Meizhou People’s Hospital, Meizhou, China; ^3^Guangdong Provincial Key Laboratory of Precision Medicine and Clinical Translation Research of Hakka Population, Meizhou, China; ^4^Key Laboratory of Tropical Disease Control, Sun Yat-sen University, Ministry of Education, Guangzhou, China

**Keywords:** *Acinetobacter baumannii*, carbapenem resistance, transcription profile, capsule synthesis, biofilm formation

## Abstract

37662RM1 and 37662RM2 are two phenotypically different, carbapenem-resistant mutants of *Acinetobacter baumannii* 37662 isolate following selection with meropenem (MEM) at sub-inhibitory concentrations. 37662RM2 lacks capsule synthesis and shows dramatically increased biofilm formation, while 37662RM1 shows merely impaired capsule synthesis. Here we report that 37662RM1 and RM2 have transcription profiles that are different from those of their starting strain, 37662WT. There were far more differentially expressed genes in 37662RM2 than in 37662RM1. The capsule polysaccharide (CPS) synthesis-required genes (*itrA2*, *gtr5*, *psaA*, *psaB*, *psaC*, *psaD*, *psaE*, *psaF*, *kpsS2, wzx*, *wzy*, *wza*, *wzb*, and *wzc*) showed reduced transcription levels in 37662RM2, which may at least partially explain the loss of capsule synthesis. The *csu* operon genes responsible for pili assembly and their regulator genes *bfmR*-*bfmS* were over-expressed in 37662RM2. This result together with the established critical roles of these genes in biofilm formation provide solid evidence that up-regulation of *csu* and *bfmR*-*bfmS* should be considered responsible for the enhanced biofilm formation in 37662RM2. IS*Aba*1 was found to insert into the intergenic region between the *csu* operon and the *acrR* gene and should be responsible for the significant up-regulation of *acrR*, which was proposed to be associated with biofilm formation. Genome sequencing revealed that the IS*Aba*1 upstream *bla*_OXA–__508_ (a new member of *bla*_OXA–__51_-like) and *acrR* were duplicated, suggesting a replicative transposition event. Altogether, the phenotype divergence driven by MEM selection mainly occurs at the RNA level and the transposition of IS*Aba*1 plays an important role in modulating gene expression to adapt to the environment.

## Introduction

Previous studies have shown that some antibiotics at sub-inhibitory concentrations could change bacterial transcription patterns and subsequent phenotypes (Goh et al., 2002; Bernier and Surette, 2013). This phenomenon was supposed to be related to activation or inactivation of the genes involved in metabolism process and regulation, resistance, virulence, DNA repair, and stress responses (Goh et al., 2002; Blazquez et al., 2012). Occurrence and patterns of these changes seem to be variably affected by antibiotics type, concentration, and action mechanism as well as bacterial species and environment (Hoffman et al., 2005; Baharoglu and Mazel, 2011; Kaplan et al., 2012). Nonetheless, the details of the influence of different antibiotics on different bacteria and the underlying mechanisms remain largely unknown.

*Acinetobacter baumannii* is among the most common nosocomial pathogens. Its high incidence of multidrug resistance has drawn therapeutic concern (Maragakis and Perl, 2008). One important coping strategy for multidrug-resistant *A. baumannii* infections is the use of carbapenems, primarily imipenem (IPM), and meropenem (MEM). *A. baumannii* 37662 is a carbapenem-sensitive isolate. After selection with sub-inhibitory concentration of MEM, two carbapenem-resistant mutants with distinct phenotype were produced, designated as 37662RM1, and 37662RM2 (Chen et al., 2017). Although both mutants share the same resistance mechanism, forming IS*Aba*1-*bla*_OXA–__508_ through IS*Aba*1 transposition to upstream of *bla*_OXA–__508_, a new member of *bla*_OXA–__51_-like, they showed differences in growth rate, colony morphology, biofilm formation, and capsule synthesis (Chen et al., 2017). Unlike the wild type strain 37662WT, 37662RM1 impaired capsule synthesis moderately while 37662RM2 abolished capsule synthesis and showed markedly enhanced biofilm formation (Chen et al., 2017). Nonetheless, the underlying mechanisms remain unknown. Whether these two mutants have other underlying transcriptional changes deserves further investigation. This is critical for evaluating the effect of MEM on *A. baumannii* in detail.

Capsule polysaccharide (CPS) and biofilm not only provide protection from phagocytosis, desiccation, and the antimicrobial effects of antibiotics, but also function as important virulence factors (Geisinger and Isberg, 2015; Harding et al., 2018). Both CPS synthesis and biofilm formation are complicated processes, although they are not well characterized in *A. baumannii*. *In silico* analysis combined with experimental results revealed that multiple genes function in coordination to produce CPS (Hu et al., 2013; Lees-Miller et al., 2013). CPS synthesis begins with the formation of undecaprenol phosphate (Und-P)-attached monosaccharide on the cytoplasmic face of the inner membrane, involving *itrA2* (encoding an initiating glycosyltransferase), followed by an ordinal synthesis of Und-P-attached oligosaccharide under the roles of a series of genes encoding glycosyltransferases and other enzymes responsible for nucleotide-sugar biosynthesis (Kenyon and Hall, 2013; Kenyon et al., 2014; Arbatsky et al., 2016). After translocation into the periplasm under the role of the translocase encoded by *wzx*, the oligosaccharide units are polymerized to form CPS by *wzy* (Lees-Miller et al., 2013). Finally, CPS moves across the outer membrane through the channel formed by the protein products of *wza*, *wzb*, and *wzc* and links to the outer surface of the cell (Hu et al., 2013). Comparison of 10 complete *A. baumannii* genome sequences revealed that the majority of the genes involved in the CPS synthesis are clustered in a locus flanked by *fkpA* and *lldP*, designated as K locus. Although the gene composition and arrangement showed variation in different *A. baumannii* strains, two highly conserved modules exist, i.e., capsule export gene module (including *wza*, *wzb*, and *wzc*) and simple sugar synthesis gene module (mainly including *galU*, *ugd*, *gpi*, *gne1*, and *pgm*) (Kenyon and Hall, 2013).

A wide range of bacterial factors and environmental cues are involved in biofilm formation. The process of surface attachment and biofilm development and maturation of *A. baumannii* on abiotic surfaces was proposed based on the findings of many studies (Stanley and Lazazzera, 2004; Gaddy and Actis, 2009; Anbazhagan et al., 2012). *Pili* production completed by the CsuA/BABCDE usher-chaperone assembly system plays an essential role in the attachment of planktonic cells to the abiotic surface, resulting in colony formation, and subsequently the full development of biofilm. The *csu* operon is regulated by a two-component system *bfmS*/*bfmR* (encoding a sensor kinase and a response regulator, respectively), whose inactivation inhibited the expression of *csu* and the formation of biofilm (Longo et al., 2014). The intercellular adhesion and biofilm maturation also require the presence of the proteins Bap (biofilm-associated protein), OmpA (the outer membrane protein of 38 kDa), AbaI (an autoinducer synthase, part of the quorum sensing QS system), and PgaA/B/C/D (responsible for the synthesis of poly-β-1,6-N-acetylglucosamine, a major component of the biofilm exopolysaccharidic matrix) (Longo et al., 2014).

Whether and how the genes essential to CPS synthesis and biofilm formation changed are critical for elucidating the mechanisms underlying changes in CPS and biofilm in 37662RM1 and RM2. Here we analyzed the transcription profiles of 37662WT, RM1, and RM2 through RNA-sequencing and characterized the differentially expressed genes. The RNA levels of the genes required for CPS synthesis and the genes required for biofilm formation were measured and compared. An IS*Aba*1 insertion into intergenic region between the *csu* operon and the *acrR* gene in 37662RM2 was revealed by genome sequencing. The insertion site sequences of all IS*Aba*1 found in 37662 strains were aligned and summarized.

## Materials and Methods

### Bacterial Strains

*Acinetobacter baumannii* 37662 and its carbapenem-resistant mutants 37662RM1 and 37662RM2 have been described previously (Chen et al., 2017). Briefly, *A.baumannii* 37662 was selected with 0.5 × minimum inhibitory concentration of MEM for 35 days, with daily passage. Two morphologically different mutants were obtained, i.e., 37662RM1 (big and opaque) and 37662RM2 (small and translucent). Their phenotype and resistance to carbapenem were confirmed to be very stable.

### RNA Isolation and RNA Sequencing (RNA-Seq) Analysis

*Acinetobacter baumannii* 37662WT and its mutants RM1 and RM2 were grown to log phase in Luria-Bertani (LB) broth with shaking at 37°C and then subjected to RNA isolation with TRI Reagent (Sigma-Aldrich) according to the manufacturer’s instructions. RNA quality and concentration were determined using an Agilent 2200 TapeStation system (Agilent Technologies, Santa Clara, CA, United States) and NanoDrop 2000 Spectrophotometer (Thermo Fisher Scientific, Waltham, MA, United States), respectively.

RNA-seq was carried out using an Illumina HiSeq 2500 (Illumina, Santiago, CA, United States) by RiboBio (Guangzhou, China) as described previously (Peng et al., 2017). Briefly, libraries for RNA-seq were prepared, purified, assessed, and subsequently sequenced with 2 × 100 bp paired-end reads. The gene expression abundance was normalized by FPKM (fragments per kilobase of exon per million fragments mapped) (v2.2.1) (Dong et al., 2017). Differentially expressed genes (DEGs) were identified, with the threshold |log2(Fold change)| > 1 and *q*-value < 0.05 as the criteria of significant gene expression difference. The transcriptional responses of 37662RM strains were summarized and the differentially expressed genes were arranged according to their locus tag. The RNA-seq data was submitted to sequence read archive (SRA) and assigned accession number PRJNA548006.

Enrichment of the differentially expressed genes was determined through gene ontology (GO) analysis based on the identified DEGs by performing the MIPs functional catalog (Dong et al., 2017). The differentially expressed genes were divided into three categories, i.e., biological processes, molecular function, and cellular components.

### Reverse Transcription-Quantitative PCR (RT-qPCR)

Total RNAs were isolated from 37662WT and its mutants by using TRI Reagent (Sigma-Aldrich, United States). The mRNA levels of the target genes were measured by RT-qPCR as described previously and normalized to that of 16S rRNA gene (Zhou et al., 2015). Three experiments were carried out independently. All the required primers in this study are listed in Supplementary Table S1. The PCR products were verified by sequencing.

### Genome Sequencing and Assembly

The genomic DNAs were isolated from the strains 37662WT, 37662RM1, and RM2 as described previously (Traglia et al., 2018). Whole genome sequencing was carried out by using Illumina GA II (Illumina Inc., United States). The raw short-read sequences were assembled *de novo* using short oligonucleotide alignment program (SOAP) (Luo et al., 2012) and the single base accuracy of the assembled genome sequences was evaluated using SOAP aligner through the service provided by BGI-Shenzhen (Li et al., 2009). The genome sequencing data was submitted to SRA and assigned accession number PRJNA545609.

### 5′-Rapid Amplification of cDNA Ends (5′-RACE)

Total RNAs were isolated from 37662WT and its mutants by using TRI Reagent (Sigma-Aldrich, United States), following digestion with DNase I (Ambion, Thermo Fisher Scientific, United States) at 37°C. 5′-RACE PCR was performed by using a RACE-kit according to the manufacture’s instruction (Takara, Dalian, China). Briefly, total RNA was reverse-transcribed for first strand cDNA synthesis with the random primer; the cDNA was then used for PCR with a forward primer annealing to the 5′-end adaptor provided by the kit and a reverse primer (5′-aacgtcaacctgtccagtaacagctgc-3′) specific to *csuA/B*. Two PCR controls with only one of the primer pair was performed to exclude unspecific amplification. The amplified PCR fragments was separated by agarose electrophoresis and subjected to sequencing.

### Statistical Analysis

The differences in expression of CPS and biofilm associated genes between 37662WT and its mutants were analyzed using Student’s *t* test and considered significant difference with the *P* value below 0.05. The data entry and analyses were performed using the statistical package for the social science (SPSS) software version 22.0 (SPSS Inc., Chicago, IL, United States).

## Results

### Characterizing the Genes Differentially Expressed in 37662RM1 or 37662RM2

**FIGURE 1:**
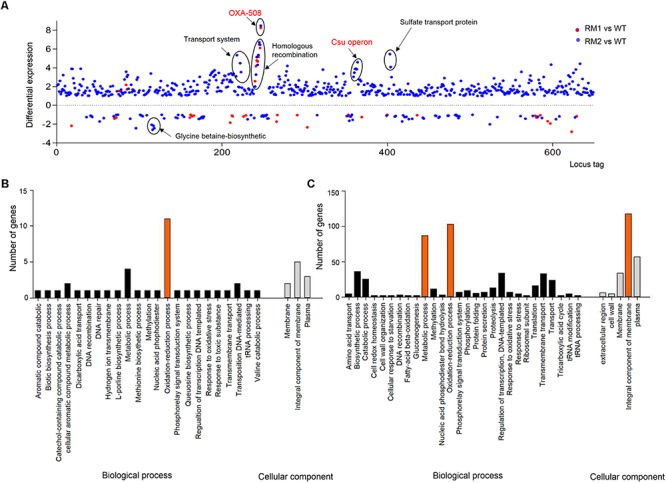
Overview of the transcriptional differences among 37662WT, 37662RM1, and 37662RM2. **(A)** The genes differentially expressed in 37662RM1 and 37662RM2 are shown in red and blue dots, respectively. The dots indicate the differential expression of all open reading frames, sorted on the *x*-axis according to the locus tag. The differential expression level is represented by (log_2_ transformed fold change) relative to 37662WT. Genes with *P* < 0.05 are considered to be differentially expressed. Some differentially expressed genes are circled here, with KEGG pathway information given. GO enrichment analysis of the genes differentially expressed in **(B)** 37662RM1 and **(C)** RM2. The genes were grouped into two categories, biological processes and cellular components.

The genes differentially expressed in 37662RM1 or RM2 were primarily identified by comparing their respective levels of transcription to those of 37662WT following RNA-seq. A total of 50 and 643 genes showed significant alterations in expression in 37662RM1 and 37662RM2, respectively, and 28 of them were shared by both mutant strains (Supplementary Table S2). According to the transcription profiles, 12 and 38 genes were up- and down-regulated in 37662RM1, respectively; 557 and 86 genes were up- and down-regulated in 37662RM2, respectively (Figure 1A). GO analysis showed that the genes differentially expressed in 37662RM1 were enriched in oxidation reduction processes (Figure 1B). Those in 37662RM2 were enriched not only in oxidation reduction process but also in integral component of the membrane and of metabolic processes (Figure 1C). Almost all the only genes whose expression up-regulated significantly in both mutant strains were those of the *bla*_*OXA–*__508_ gene cluster (Figure 1A and Supplementary Table S2). All other genes whose expression varied dramatically were mainly found in 3766RM2 and could be grouped into four groups based on KEGG pathways analysis, including the up-regulated transport system, the *csu* operon, the sulfate transport proteins, and the down-regulated glycine betaine biosynthetic operon (Figure 1A). These results indicated that MEM at sub-MIC could induce the changes in the transcription profiles of *A. baumannii*, and most of them appear to occur randomly.

### Measuring the RNA Levels of the Genes Responsible for CPS Production

**FIGURE 2:**
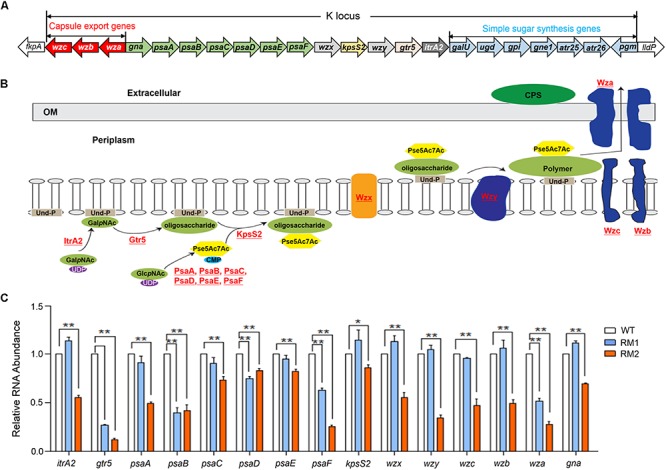
Quantification analysis of the CPS synthesis-related genes. **(A)** Schematic drawing of the K locus gene array in *Acinetobacter baumannii* 37662 genome. Horizontal arrows showed the direction of transcription, with assigned gene names shown. The genes having the similar function were marked in the same color. **(B)** Schematic representation of a brief model of CPS biosynthesis in *A. baumannii* with the detected genes shown in red and underlined. GalpNAc, N-acetylgalactosamine; Und-P, undecaprenol phosphate; GlcpNAc, N-acetylglucosamine, Pse5Ac7Ac, di-acetylpseudaminic acid. **(C)** RT-qPCR analysis of the genes closely associated with CPS synthesis in 36772WT, 36772RM1, and RM2. Their expression levels were normalized to that of 16S rRNA. Three experiments were carried out independently and the results are represented as mean ± SD. ^∗^*P* < 0.05; ^∗∗^*P* < 0.01.

The K locus sequences were extracted from the genome sequences of 37662WT and both mutants and the contained genes were identified through BLAST. The gene composition and arrangement was shown in Figure 2A. The role or potential function of these genes involved in CPS synthesis was schematically shown in Figure 2B. Most of them were not found in the RNA-seq data of 37662WT, 37662RM1, or 37662RM2 possibly due to the low abundance of their transcripts. However, RT-qPCR analysis showed that all the detected genes were down-regulated to some extent in 37662RM2 in comparison with 37662WT, while only some of them, including *gtr5*, *psaB, psaD*, *psaF*, and *wza*, showed moderate reductions in RNA levels in 37662RM1 (Figure 2C). This difference between 37662RM1 and 37662RM2 corresponds to their difference in the CPS synthesis, which was almost entirely abolished in 37662RM2 and only moderately impaired in 37662RM1. For this reason, the decreased expression of the CPS synthesis genes, including but not limited to *itrA2*, *gtr5*, *psaA*, *psaB*, *psaC*, *psaD*, *psaE*, *psaF*, *kpsS2*, *wzx*, *wzy*, *wza*, *wzb*, and *wzc* genes should be, at least partially, responsible for the capsule loss of *A. baumannii* 37662RM2. K locus sequence comparison revealed a C > T substitution occurred at position 607 within the *gne2* open reading frame of 37662RM2, resulting in P203S amino acid change, while the sequences of other K locus genes and flanking regions were completely identical in 37662 WT, RM1, and RM2. The effect of this mutation on CPS synthesis is not clear yet, meanwhile, the reason for the down-regulation of these genes required for CPS synthesis remains elusive.

### Detecting the Genes Involved in Biofilm Formation

**FIGURE 3:**
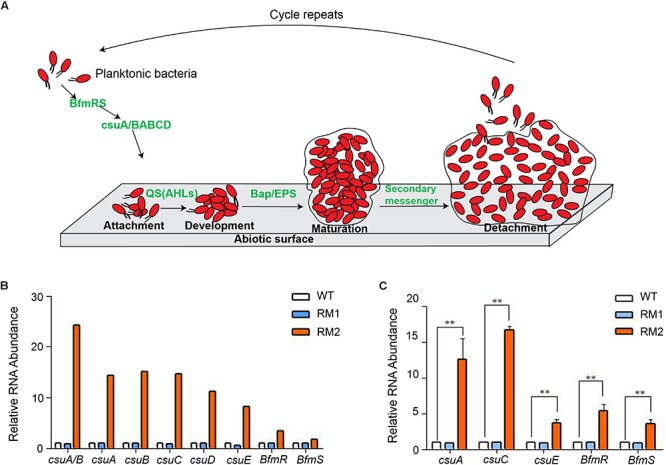
Quantification analysis of the *csu* operon. **(A)** Schematic representation of a brief model of *A. baumannii* forming biofilm on abiotic surfaces. Pilus is produced by the CsuA/BABCDE usher-chaperone and mediates attachment of planktonic cells to the abiotic surface, resulting in colony formation. High population density induces microbes to secrete autoinducer molecules, which turns on the N-acyl-homoserine lactone (AHL) type of quorum-sensing system to promote biofilm development (Anbazhagan et al., 2012). Microcolonies transition to mature form by multiplication and secretion of polymeric substances, the bacteria biofilm-associated protein (Bap), and extracellular polymeric substances (EPS) (Flemming et al., 2007). Biofilm can be dispersed in clumps and individual forms from the attached surface under oxygen- and nutrient-depletion condition, presence of toxins, and secondary messenger molecule cyclic-di-GMP switching the sessile form to planktonic form (Sagar et al., 2016). **(B)** RNA-seq data showing the expression of the *csu* operon genes and the regulating genes *bfmR* and *bfmS* in 37662 WT and mutants. **(C)** RT-qPCR analysis of the *csu* operon genes as well as *bfmR* and *bfmS.* Their expression levels were normalized to that of 16S rRNA. Three experiments were carried out independently and the results are here represented as mean ± SD. ^∗∗^*P* < 0.01.

Multiple genes are involved in bacterial biofilm formation as shown in Figure 3A. Among these, the *csu* operon genes, required for pilus synthesis and assembly, play a critical role in biofilm formation. RNA-seq data showed that all the genes of the *csu* operon, and its regulator *bfmS*-*bfmR*, were significantly up-regulated in 37662RM2 but not in 37662RM1, corresponding to the enhanced biofilm formation in 37662RM2 (Figure 3B). In contrast, the RNA levels of other genes participating in biofilm formation, including but not limited to N-acyl-homoserine lactone (AHL) type of quorum-sensing system, *bap* and *blp*, changed little in 37662RM2 revealed by RNA-seq (Supplementary Table S3). RT-qPCR analysis confirmed that *csuA*, *csuC*, *csuE*, *bfmR*, and *bfmS* up-regulated in 37662RM2 but weakly down-regulated or unchanged in 3766RM1 (Figure 3C), while *bap* and *blp* just changed weakly or not at all in 37662 mutants (Data not shown). These results together with the established critical roles of these genes in biofilm formation (Longo et al., 2014) indicated that up-regulation of *csu* and *bfmR*-*bfmS* should be considered responsible for the enhanced biofilm formation in 37662RM2.

### Evaluating the Influence of IS*Aba*1 Transposition on *acrR* Expression

**FIGURE 4:**
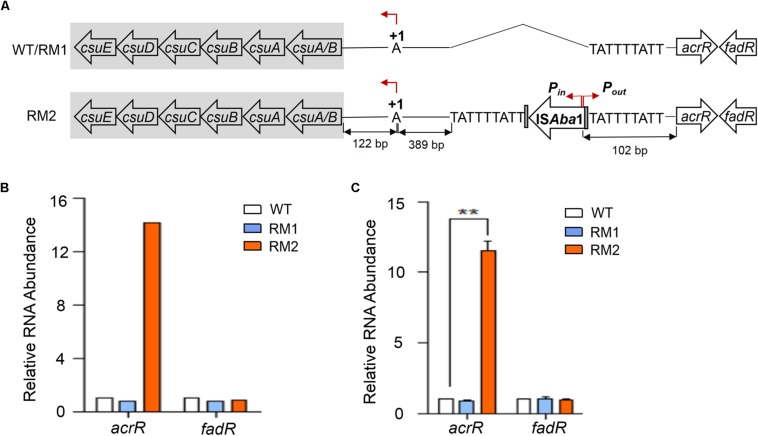
Quantification analysis of the *acrR* gene. **(A)** Schematic representation of the *csu*-IS*Aba*1-*acrR* gene array. Horizontal arrows showed the direction of transcription, with assigned gene names shown. +1 represents the transcription stat site of *csu*. IS*Aba*1 contains two over-lapped, divergently oriented promoters, with *P*_*in*_ transcribing transposase genes inside the IS*Aba*1 element, *P*_*out*_ transcribing adjacent genes outside IS*Aba*1. **(B)** The expression of *acrR* and its downstream gene *fadR* in RNA-seq of 37662WT and mutants. **(C)** RT-qPCR analysis of *acrR* and *fadR*. Their expression levels were normalized to that of 16S rRNA. Three experiments were carried out independently and the results are represented as mean ± SD. ^∗∗^*P* < 0.01.

Whole genome sequencing data comparison showed that an IS*Aba*1 element was inserted into the intergenic region between the *csu* operon and the *acrR* gene in 37662RM2 but neither in 37662WT nor in 37662RM1. As shown in Figure 4A, this IS is flanked by a 9 bp duplicated target site sequence TATTTTATT at both sides, 511 and 61 bp away from the *csuA/B* and *acrR* start codon, respectively; the transcriptional orientation of IS*Aba*1 transposase genes was identical to that of *csu* and opposite to that of *acrR*; the IS*Aba*1-intrinsic outward-directed promoter (*P*_out_) was located 102 bp upstream of the start codon of the *acrR* gene. This result strongly suggested that IS*Aba*1-*P*_out_ could initiate the expression of the *acrR* gene. As expected, the RNA level of *acrR* increased significantly up to 14-fold in 37662RM2 revealed by RNA-seq data, while the RNA level of the downstream gene *fadR* with an opposite transcriptional orientation changed little (Figure 4B), which was then confirmed by RT-qPCR analyses (Figure 4C). Altogether, these results suggest that MEM selection could induce IS*Ab*a1 transposition and the insertion of IS*Aba*1 should be responsible for the up-regulation of the *acrR* gene in 37662RM2 by providing the built-in promoter *P*_out_.

### Characterizing the Insertion Site Sequences of IS*Aba*1 in *A. baumannii* 37662

**FIGURE 5:**
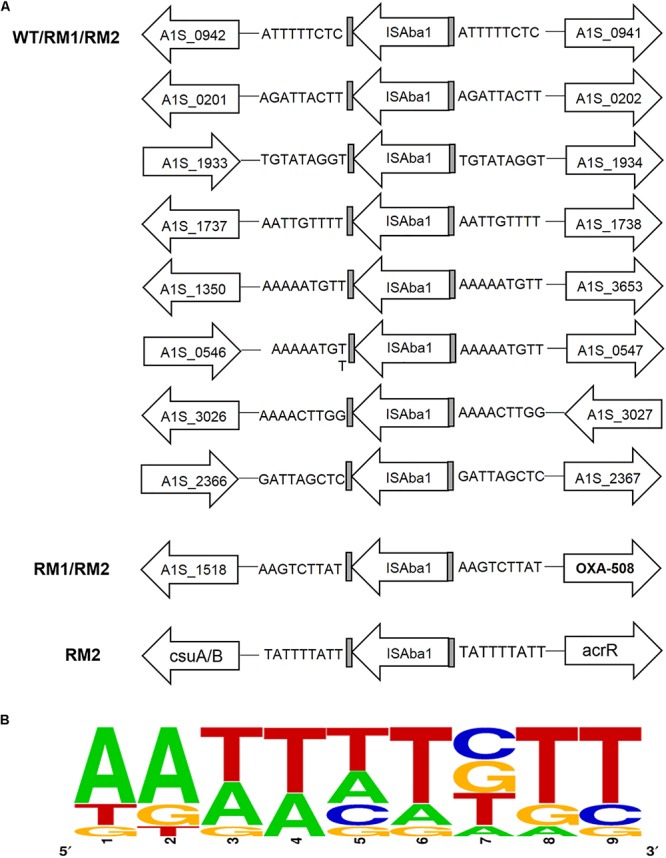
Whole-genome sequence analysis of IS*Aba*1 insertion sites sequences in 37662 strains. **(A)** Schematic showing all the gene locus containing IS*Aba*1 in 37662WT and RM strains, with duplicated target site sequences shown. **(B)** The consensus pattern of IS*Aba*1 insertion site sequences generated by using Weblogo online software (http://weblogo.berkeley.edu/logo.cgi).

Insertion of IS*Aba*1 is always accompanied by duplication of the insertion site sequence. To identify the potential transposition site of IS*Aba*1, IS*Aba*1 was searched in the whole genome sequencing data of 37662WT, RM1 and RM2, and the duplicated target sequences adjacent to IS*Aba*1 were characterized. A total of 10 IS*Aba*1 were found: 8 of which are common in 37662WT and the two mutants; one was shared by the two mutants and located upstream of *bla*_OXA–__508_; and the residual one was unique in 37662RM2 and located upstream of the *csu* operon (Figure 5A). These results suggested that the additional IS*Aba*1 in both mutants might be generated through a replicative pathway during the transposition event but not excised or transferred from other sites. Sequence analysis showed that each of the 10 IS*Aba*1 was flanked by a duplicated 9 bp sequence. Two of them shared the same sequences but all the others were different (Figure 5A). These insertion site sequences were highly AT-rich with a consensus sequence AA (A/T) (A/T) WTWTT (W is G, C, T, or A) (Figure 5B), a little bit different from the previous reported AAATAAATT consensus motif (Mugnier et al., 2009). Notably, the AAATAAATT consensus motif was deduced from a total of 24 IS*Aba*1 insertion site sequences, however, the sequences showed significant variability (Supplementary Figure S1). More insertion site sequences of IS*Aba*1 need to be found and characterized to pave the way for the prediction of IS*Aba*1 insertion sites.

## Discussion

A growing consensus has emerged that sub-inhibitory concentration of some antibiotics possess biological activities and can alter the bacterial transcription and translation patterns (Davies et al., 2006). Our previous (Chen et al., 2017) and present studies showed that 37662RM1 and RM2, two mutants of *A. baumannii* 37662 produced after MEM selection, changed their protein and transcription profiles, which is consistent with previous observations that the resistant *A. baumannii* mutants showed different gene expression profiles after imipenem exposure (Chang et al., 2014). MEM might promote the transposition of IS*Aba*1 in *A. baumannii* 37662 and caused the formation of IS*Aba*1-*bla*_OXA–__508_ (Chen et al., 2017) and IS*Aba*1-*acrR* herein, resulting in transcription activation of *bla*_OXA–__508_ and *acrR*, respectively. These results provide solid evidence that carbapenems can act as bioactive molecules and change the expression profiles of *Acinetobacter* spp. at transcriptional level. Here, 50 and 643 genes were found to be differentially expressed in 37662RM1 and RM2, respectively, and only 28 genes were shared among the two mutants (Figure 1A and Supplementary Table S2). In another report, 88 and 68 genes differentially expressed in IPM-2 m and IPM-8 m, which are two resistant mutants produced from *A. baumannii* ATCC17978 after selection with imipenem at concentrations of 0.5 and 2 μg/ml, respectively; only 38 genes were common to both mutants (Chang et al., 2014). Noticeably, even genes that were altered in both mutants sometimes changed in the opposite direction between 37662RM1 and RM2 (Supplementary Table S2) as well as between IPM-2 m and IPM-8 m (Chang et al., 2014). Further comparison revealed that up-regulation of the *bla*_OXA–__51_-like gene cluster is the only similarity in all the differentially expressed genes from mutants 37662RM1, RM2, IPM-2 m, and IPM-8 m (Supplementary Table S4), corresponding to the carbapenem selection and resistance production. The highly variable changes in transcriptional profiles are assumed to depend on antibiotics, bacterial species, nutrients, and other environmental conditions (Baharoglu and Mazel, 2011). Considering that 37662RM1 and RM2 originated from the same strain in an identical environment, the similarities and large differences in transcription profile between these two carbapenem-resistant mutants drove us to propose that sub-inhibitory concentration of carbapenem could promote *A. baumannii* to produce variable mutations to adapt to environmental changes and other random mutations as a response to antibiotic effects. Correspondingly, the expression of some genes involved in SOS response or DNA repairs up-regulated in 37662 mutants revealed by RNA-seq data (Supplementary Table S5).

MICs of MEM changed from 1 μg/ml against the strain 37662WT to 16 μg/ml against the mutants 37662RM1 and RM2 (Chen et al., 2017). This increase was mainly due to the insertion of an IS*Aba*1 into upstream of *bla*_OXA–__508_ and to the over-production of OXA-508 (Chen et al., 2017). It has been recognized that acquisition and expression of plasmid-carrying carbapenemase genes is the major mechanism responsible for carbapenem resistance in *A. baumannii* (Poirel and Nordmann, 2006). Several other mechanisms have also been described, including efflux pump over-expression, porin down-regulation, and penicillin-binding protein alterations (Poirel and Nordmann, 2006). To determine whether other mechanisms contributed to the carbapenem resistance, the transcription level of carbapenem resistance-related efflux pump genes, porin genes, and penicillin-binding protein coding genes of 37662RM1 and RM2 were compared to those of the starting strain 37662WT based on the RNA-seq data. As shown in Supplementary Table S6 (marked with ^∗^), the transcripts levels of an RND-type efflux pump gene (A1S_0010), some putative RND type efflux pump genes, drug transporter genes, and multidrug efflux pump genes increased moderately in 37662RM2, consistent with the weak effect of addition of PaβN, the RND-type efflux pump inhibitor, on the MIC values of carbapenem against 37662RM1 and RM2 (Chen et al., 2017). In addition, the RNA level of *oprC* increased in 37662RM2, consistent with the previous protein profile analysis showing the accumulation of OprC proteins in 37662RM2 (Chen et al., 2017).

Multiple genes are expressed to orchestrate the biofilm formation, among which, the *csu* operon plays a critical role. Our results showed that the strong biofilm formation ability of 37662RM2 was mainly due to the up-regulation of the *csu* operon and its regulator gene *bfmR*/*bfmS*, while the expression of other genes involved in the biofilm formation process, such as quorum sensing and secretion systems, changed little. Considering that up-regulation of *bla*_OXA–__95_ and down-regulation of the *csu* operon occurred in two different mutants from two different selections, Chang et al. (2014) suggested that *A. baumannii* shows an inverse relationship between carbapenem resistance and biofilm production. Nonetheless, our data indicated that carbapenem resistance is not essentially related to down-regulation of the *csu* operon or the subsequent decrease in biofilm production. The carbapenem-resistance and up-regulation of the *csu* operon and enhancement of biofilm formation occurred simultaneously in 37662RM2; while in 37662RM1, carbapenem resistance was observed but the *csu* expression and biofilm formation changed little. In addition, some other studies also showed that sub-inhibitory concentration of imipenem and aminoglycoside antibiotics induced biofilm formation in *A. baumannii* and *Pseudomonas aeruginosa*, respectively (Hoffman et al., 2005; Nucleo et al., 2009). Based on the previous studies reporting that CPS plays a critical role in restricting the continual growth of mature biofilm (Joseph and Wright, 2004), we assumed that the enhanced biofilm formation would be closely related to the loss of CPS in 37662RM2. As a critical gene required for CPS synthesis, *itrA2* is also involved in the biofilm formation in *A. baumannii*. Deletion of *itrA2*, i.e., *pglC* (Δ*pglC*), caused the change and rearrangement of the biofilm structure revealed by scanning electron microscope, however, the total biofilm mass was not affected (Lees-Miller et al., 2013). Herein, down-regulation of the *itrA2* gene and up-regulation of the *csu* operon occurred simultaneously in 37662RM2. Although the overall mass of biofilm increased significantly, the potential effect of *itrA2* down-regulation on biofilm structure and arrangement merits investigation to unveil the detailed relationship between CPS synthesis and biofilm formation.

37662RM2 contains an unique IS*Aba*1 transposition in contrast to 37662WT and RM1, which occurred in the region between *csuA/B* and *acrR* and enhanced the transcription of *acrR* by providing IS*Aba*1-*P*_*out*_ (Figure 4A). While IS transcription efficiency is affected by multiple factors, including but not limited to host factors, strain, IS types, chromosome structure, and growth conditions (Ellis et al., 2015; Vandecraen et al., 2017), a consensus is growing that IS transposition to AT-rich region is a random event. Interestingly, two other independent cases of IS*Aba*1 insertion between *csuA/B* and *acrR* was also found in *A. baumannii* by a BLAST search in NCBI, with accession No. CP016300 and CP021782, while the expression of the neighboring genes was not reported by the submitter. These results may imply a potential modulation of IS*Aba*1 on neighboring genes and the subsequent bacterial adoption to environment. AcrR, belonging to the TetR family, is a transcriptional regulator and may have an inhibitory role on the expression of the *acrAB* operon (Subhadra et al., 2018). AcrAB efflux pump is reported to be associated with biofilm formation (Kvist et al., 2008), however, *acrAB* of 37662RM2 displayed little difference in expression from that of 37662WT and RM1 revealed by RT-qPCR (data not shown). Therefore, the function of AcrR and the effect of *acrR* up-regulation on bacterial physiological behaviors need further investigation.

Given that BfmR/BfmS plays a positive role in regulating the expression of the *csu* operon, increased expression of *bfmR* and *bfmS* contributed to the significant up-regulation of the *csu* operon in 37662RM2 (Figures 3B,C). Sequence comparison showed that IS*Aba*1 insertion is the only difference within the *csu-acrR* gene cluster between 37662RM2 and the other two strains. Several known mechanisms are responsible for IS-mediated activation of neighboring genes, including IS insertion providing a complete outward-directed promoter or forming a hybrid promoter, read-through transcription from the IS transposase promoter, causing DNA topological changes, as well as several other not determined mechanisms (Vandecraen et al., 2017). The significant *csu* up-regulation in 37662RM2 was not related to IS*Aba*1-*P*_out_. To detect whether the *csu* up-regulation was caused at least in part by co-transcription mediated by the promoter *P*_in_ of the IS*Aba*1 transposase gene (Mugnier et al., 2009), the transcription start site (TSS) of *csu* was mapped via 5′-RACE PCR. The result showed that 37662 and its two mutants shared the same TSS (Figure 4A), indicating an inexistence of co-transcriptional activation for *csu* expression in 37662RM2.

In summary, sub-inhibitory concentration of MEM changed the transcriptional profiles of *A. baumannii* in various ways and thus caused differentiated phenotypic, physiological, and metabolic changes in different mutants. The relationship between antibiotics and IS transposition merits further investigation to fully determine the mechanisms underlying antibiotics and their effect on bacteria.

## Data Availability Statement

The datasets generated for this study can be found in the accession numbers of RNA-seq data and genomic data are PRJNA548006 and PRJNA545609, respectively.

## Author Contributions

QG, XM, and XG designed and performed the research, and wrote the manuscript. HG, XC, HY, and YQ performed the statistical analyses. All authors read and approved the final manuscript.

## Conflict of Interest

The authors declare that the research was conducted in the absence of any commercial or financial relationships that could be construed as a potential conflict of interest.
