# Supplementary material for: Two Phenotype-Differentiated Acinetobacter baumannii Mutants That Survived in a Meropenem Selection Display Large Differences in Their Transcription Profiles
Source: Front Microbiol. 2019 Oct 9;10:2308. doi: 10.3389/fmicb.2019.02308 (PMC6794425; doi:10.3389/fmicb.2019.02308)
Supplement: Supplementary file 1 [file Data_Sheet_1.doc]

**Table S1 |** RT-qPCR primers used in the present study.

| **Primer name** | **Primer sequence (5’-3’)** | **PCR products** | **Reference** |
| --- | --- | --- | --- |
| itrA2-F | TAACTGTTCTGTCGCCAA | *itrA2* | This study |
| itrA2-R | CCAGGTCTTTCTTGGTAA |
| psaF-F | TGTGTTACAACTTCGCAG | *psaF* | This study |
| psaF-R | CCGTGGTAACATTGTCTC |
| gtr5-F | AGTAATTGCTCACTGGCA | *gtr5* | This study |
| gtr5-R | GCCTTTCCTGTACCTACA |
| psaE-F | AGATTAAGCAAGCCTACC | *psaE* | This study |
| psaE-R | ATACGTTCCAGGTGATTG |
| psaB-F | TGTTCATGTCTGTGGTGG | *psaB* | This study |
| psaB-R | ACAACTCGGTTACCAACA |
| psaC-F | TCGTATGTGGGTGCTAAG | *psaC* | This study |
| psaC-R | GTGCAACTGTTTCAGCAG |
| psaA-F | TGGTGCT GCTGGTTATAT | *psaA* | This study |
| psaA-R | TCCATAATCCCAACAGAG |
| gna-F | GGATATGTAGGCTTGCCA | *gna* | This study |
| gna-R | AAGCTCTTCTGGAGAAAC |
| psaD-F | AGCCGTGTCAGTCCTAGT | *psaD* | This study |
| psaD-R | AGCTACTGTCAGAATACC |
| kpsS2-F | AACAGGAGTAGAGATTGG | *kpsS2* | This study |
| kpsS2-R | GAACTACAGGAGAATCTG |
| wzx-F | TGTACGTTCTGGTGGTTGG | *wzx* | This study |
| wzx-R | TACAGCCAAAGTACCGAGC |
| wzy-F | GGCTTCTTATGCACCTAGTTG | *wzy* | This study |
| wzy-R | CCAGTAAACCGTACCCCTGC |
| wza-F | AACGGACCTCGGAACTAC | *wza* | This study |
| wza-R | AATGATGCGTAGTCGCGT |
| wzb-F | CGTAGCCCTATGGCAGAA | *wzb* | This study |
| wzb-R | CATCGGCTTGATGCCCAA |
| wzc-F | ACGCCAGATACCTATTCG | *wzc* | This study |
| wzc-R | TCTGCTTGAGCCGGTGAT |
| csuA-F | TTGGTGAAGCTACCACAG | *csuA* | This study |
| csuA-R | TACCAGCACACTCGATCT |
| csuC-F | ACCTGTGATGGCACAAGC | *csuC* | This study |
| csuC-R | CACCATCGCATCGGTCTT |
| csuE-F | TGGACTGATGTTGACAGG | *csuE* | This study |
| csuE-R | ATAGGTCATGTTCGCCAA |
| bfmR-F | GAGCGTTTAGCGCGATTA | *bfmR* | This study |
| bfmR-R | TCCAAGACCACAAGATCC |
| bfmS-F | AGTGAAGGAGTCGCTCGA | *bfmS* | This study |
| bfmS-R | GAGACAGCTCAACCTTAC |
| blp-F | GCTACAGGACAGACCACT | *blp* | This study |
| blp-R | ACTGTTCCTGCACCAGCT |
| bap-A1S_2724-F | TAGTGATGCGGACGCAGA | *bap* | This study |
| bap-A1S_2724-R | TAGCAGCCCATAACCATG |
| acrR-F | CCGGATTTAGTATGAGAGGA | *acrR* | This study |
| acrR-R | TGTGATGATCGGTTGCTCTA |
| fadR-F | CCTGAGCTTGTTCAAGGT | *fadR* | This study |
| fadR-R | AAGTACCCAGCCCCTGTT |
| 16S rRNA-F | CAGCTCGTGTCGTGAGATGT | *16S rRNA* | (Kuo et al., 2012) |
| 16S rRNA-R | CGTAAGGGCCATGATGACTT |

**Table S2 |** Differentially expressed genes shared in 37662RM1 and 37662RM2.

| **Locus tag** | **log_2_ (fold change) in 37662RM1** | **log_2_ (fold change) in 37662RM2** | **Protein name** |
| --- | --- | --- | --- |
| A1S_0103 | -2.19 | 3.89 | 3-hydroxyisobutyrate dehydrogenase |
| A1S_0568 | -1.31 | -1.01 | Pyridine nucleotide transhydrogenase beta subunit |
| A1S_0677 | 1.50 | 1.01 | Transposase |
| A1S_0736 | 2.19 | 3.58 | Hypothetical protein |
| A1S_0737 | 1.70 | 3.04 | 5-methyltetrahydropteroyl triglutamate-homocysteine Methyltransferase |
| A1S_1214 | -1.31 | 1.41 | Benzoate 12-dioxygenase beta subunit |
| A1S_1215 | -1.01 | 1.97 | Benzoate 12 dioxygenase alpha subunit |
| A1S_1233 | -1.09 | 2.59 | Hypothetical protein |
| A1S_1383 | -1.85 | 2.69 | Surface antigen |
| A1S_1385 | -1.10 | 2.48 | Hypothetical protein |
| A1S_1386 | -1.17 | 3.25 | Catalase |
| A1S_1511^a^ | 2.57 | 2.01 | Biotin synthase |
| A1S_1512^a^ | 4.17 | 3.27 | Putative ferredoxin |
| A1S_1513^a^ | 4.80 | 5.14 | Putative membrane protein |
| A1S_1514^a^ | 4.67 | 4.32 | Holliday junction nuclease |
| A1S_1515^a^ | 5.16 | 5.36 | XRE-family HTH |
| A1S_1516^a^ | 6.48 | 6.73 | Putative antibiotic resistance |
| A1S_1517^a^ | 8.26 | 8.49 | β-lactamase OXA-508 |
| A1S_1697 | -1.03 | 1.97 | Putative transcriptional regulator |
| A1S_1845 | -1.04 | 1.29 | CatA3 |
| A1S_1867 | -2.35 | 2.05 | General substrate transporter: Major facilitator superfamily |
| A1S_2179 | -1.01 | 1.27 | Hypothetical protein |
| A1S_2183 | -1.20 | 1.35 | Putative signal peptide |
| A1S_2230 | -1.25 | 1.43 | Hypothetical protein |
| A1S_3632 | -1.29 | 1.65 | Hypothetical protein |
| A1S_3658 | -1.70 | 1.95 | Hypothetical protein |
| A1S_3678^a^ | 6.12 | 6.47 | Hypothetical protein |
| A1S_3797 | -1.17 | 1.29 | Hypothetical protein |

*^a^ Belonging to the bla_OXA-508_ gene cluster.*

**Table S3 |** Expression of the biofilm-associated genes in 37662RM1 and RM2 based on RNA-seq data.

*^a^ This gene was annotated as putative hemagglutinin/hemolysin-related protein in the reference strain A. baumannii 17978 in NCBI but re-annotated herein as N-terminal region of Bap based on previous stud*y *(De Gregorio et al., 2015); ^b^Not available in the reference strain A. baumannii 17978, the sequence was identified from the RNA-seq data with a software pipeline bowtie2-htseq-DEseq; ^c^AHL (N-acyl homoserine lactone)-based quorum sensing; ^d^Not detected; ^e^ Belonging to quorum sensing system.*

| **Locus tag** | **log_2_ (fold change) in 37662RM1** | **log_2_ (fold change) in 37662RM2** | **Protein name** |
| --- | --- | --- | --- |
| A1S_2696 | -0.48 | 0.74 | C-terminal region of Bap (biofilm-associated protein) |
| A1S_2724 | -0.59 | 1.52 | N-terminal region of Bap^a^ |
| NA^b^ | -0.67 | 0.56 | Blp |
| A1S_0109 | 0.79 | -0.25 | Homoserine lactone synthase^c^ |
| A1S_2042 | -0.26 | 1.2 | Possible pilus assembly protein PilW |
| A1S_1507 | -0.21 | 0.71 | Fimbrial protein |
| A1S_3168 | -0.31 | 0.35 | Possible pilus assembly protein PilW |
| A1S_0112 | ND^d^ | ND | Acyl-CoA synthetase/AMP-acid ligases II^e^ |
| A1S_0113 | ND | ND | Acyl-CoA dehydrogenase^e^ |
| A1S_0115 | ND | ND | Amino acid adenylation^e^ |
| A1S_0116 | ND | ND | RND superfamily-like exporter^e^ |
| A1S_0117 | ND | ND | Hypothetical protein^e^ |
| A1S_0118 | ND | ND | Hypothetical protein^e^ |
| A1S_2218 | 0.79 | 4.60 | CsuA/B |
| A1S_2217 | 0.95 | 3.84 | CsuA |
| A1S_2216 | 1.04 | 3.92 | CsuB |
| A1S_2215 | 0.83 | 3.87 | CsuC |
| A1S_2214 | 0.95 | 3.48 | CsuD |
| A1S_2213 | 0.15 | 3.04 | CsuE |
| A1S_0748 | -0.37 | 1.75 | BfmR |
| A1S_0749 | -0.65 | 1.41 | BfmS |

**Table S4 |** Expression of the genes differentially expressed in the carbapenem-resistance mutants IPM-2 m, IPM-8 m, 37662RM1 and 37662RM2.

| **Locus tag** | **Log_2_ (fold change) in IPM-2 m** | **Log_2_ (fold change) in IPM-8 m** | **Log_2_ (fold change) in 37662RM1** | **Log_2_ (fold change) in 37662RM2** | **Protein Name** |
| --- | --- | --- | --- | --- | --- |
| A1S_0015 | 7.74 | 8.92 | 0.23 | 0.13 | Hypothetical protein |
| A1S_0016 | 7.90 | 8.30 | 0.54 | -0.12 | Site-specific tyrosine recombinase |
| A1S_0209 | 4.76 | 4.82 | 0.06 | 0.29 | Transposase |
| A1S_0210 | 3.17 | 2.89 | -0.95 | 0.26 | Transposase |
| A1S_0623 | 8.34 | 7.98 | 0.21 | 0.04 | DNA mismatch repair enzyme |
| A1S_0626 | 6.04 | 5.06 | -0.29 | 0.61 | Hypothetical protein |
| A1S_0627 | 6.20 | 6.38 | -0.56 | 0.15 | Hypothetical protein |
| A1S_0628 | 6.93 | 6.67 | 0.48 | 0.12 | Putative transposase |
| A1S_0630 | 4.72 | 5.14 | 0.25 | 0.57 | Hypothetical protein |
| A1S_0631 | 6.12 | 5.52 | -0.56 | 0.11 | Hypothetical protein |
| A1S_0632 | 7.45 | 7.19 | 0.12 | 0.09 | DNA primase |
| A1S_0633 | 6.13 | 5.74 | -0.56 | 0.89 | Hypothetical protein |
| A1S_0634 | 5.17 | 4.93 | 0.26 | -0.05 | Hypothetical protein |
| A1S_0637 | 6.21 | 6.52 | 0.56 | 0.06 | DNA-directed DNA polymerase |
| A1S_0638 | 7.37 | 7.45 | 1.20 | -0.08 | Hypothetical protein |
| A1S_0640 | 6.91 | 6.41 | 0.25 | 0.65 | Hypothetical protein |
| A1S_0646 | 7.36 | 7.00 | 0.56 | -0.25 | IcmB protein |
| A1S_0649 | 8.52 | 8.43 | 0.89 | 0.29 | Putative phage primase |
| A1S_0650 | 7.02 | 6.46 | 0.58 | -1.02 | Conjugal transfer protein |
| A1S_0651 | 6.67 | 6.32 | 0.62 | -0.26 | TraB protein |
| A1S_0652 | 8.02 | 7.83 | -0.26 | 0.68 | Putative ferrous iron transport protein A |
| A1S_0661 | 6.22 | 5.70 | -0.68 | 0.52 | Phage integrase family protein |
| A1S_0665 | 6.79 | 6.34 | 0.33 | 0.89 | Conjugal transfer protein TrbJ |
| A1S_0666 | 7.50 | 7.16 | 0.21 | 0.56 | TrbL/VirB6 plasmid conjugal transfer protein |
| A1S_0671 | 7.89 | 6.92 | -0.87 | 0.25 | Protein tyrosine phosphatase |
| A1S_1517 | 3.79 | 8.36 | 8.26 | 8.49 | Beta-lactamase OXA-51-like |
| A1S_2554 | 5.96 | 6.16 | 0.22 | 0.58 | Putative transposase |
| A1S_0087 | -3.76 | -4.52 | 0.13 | 1.35 | Short-chain dehydrogenase/reductase SDR |
| A1S_0109 | -3.62 | -6.03 | 0.79 | -0.25 | Homoserine lactone synthase |
| A1S_0161 | -2.53 | -2.86 | 0.01 | 3.90 | MFS family transporter |
| A1S_1272 | -3.30 | -3.31 | 0.22 | 1.22 | Putative transcriptional regulator |
| A1S_1296 | -3.49 | -3.45 | 0.23 | 0.31 | Hypothetical protein |
| A1S_1308 | -2.93 | -3.06 | 0.22 | 0.03 | Hypothetical protein |
| A1S_1309 | -3.75 | -2.86 | 0.08 | 0.08 | Hypothetical protein |
| A1S_2215 | -3.14 | -4.57 | -0.26 | 3.87 | CsuC |
| A1S_2212 | -2.73 | -4.45 | 0.06 | 3.92 | CsuB |
| A1S_2218 | -3.89 | -5.88 | -0.33 | 4.60 | CsuA/B |
| A1S_2487 | -3.93 | -4.93 | -0.76 | 2.10 | Hypothetical protein |

**Table S5 | Expression of DNA damage repair genes in 37662RM1 and 37662RM2.**

| **Locus tag** | **Log2 (fold change) in 37662RM1** | **Log2 (fold change) in 37662RM2** | | **Protein Name** |
| --- | --- | --- | --- | --- |
| A1S_1389 | 0.57 | 1.46 | UmuDAb, DNA polymerase V component | |
| A1S_1388 | 0.77 | 1.52 | DdrR | |
| A1S_0186 | 0.44 | 0.52 | DinB, DNA polymerase IV | |
| A1S_3295 | 0.10 | 0.39 | UvrA, UvrABC endonuclease | |
| A1S_1962 | -0.24 | -0.01 | RecA, Recombinase A | |

**Table S6 |** Expression of the antibiotic resistance-associated genes in 37662RM1 and 37662RM2.

| **Locus tag** | **Log2 (fold change) in 37662RM1** | **Log2 (fold change) in 37662RM2** | | **Protein Name** |
| --- | --- | --- | --- | --- |
| A1S_1193 | -0.40 | -0.09 | OmpA/MotB | |
| A1S_2538 | -0.48 | 0.64 | Outer membrane protein CarO precursor | |
| A1S_1705 | 0.41 | 0.91 | AdeB | |
| A1S_1751 | 0.15 | 0.56 | AdeA membrane fusion protein | |
| A1S_1752 | -0.54 | -0.33 | AdeA membrane fusion protein | |
| A1S_1753 | -0.27 | 0.48 | AdeR | |
| A1S_1754 | -0.27 | 0.41 | AdeS | |
| A1S_1755 | -0.83 | 2.18 | AdeT* | |
| A1S_2735 | -0.13 | -0.22 | AdeI | |
| A1S_2737 | 0.03 | 0.04 | AdeK | |
| A1S_0170 | -0.22 | 2.78 | Putative outer membrane copper receptor (OprC)* | |
| A1S_0535 | -0.15 | 0.07 | Putative RND family drug transporter | |
| A1S_0537 | 0.08 | 0.55 | Putative RND family drug transporter | |
| A1S_0538 | 0.06 | 0.66 | Putative RND family drug transporter | |
| A1S_0008 | -0.21 | 1.04 | Putative RND type efflux pump* | |
| A1S_0009 | -0.28 | 2.14 | Putative RND type efflux pump* | |
| A1S_0010 | -0.33 | 2.23 | RND type efflux pump* | |
| A1S_0255 | -0.19 | 0.63 | Putative RND family drug transporter | |
| A1S_0535 | -0.15 | 0.07 | Putative RND family drug transporter | |
| A1S_0537 | 0.08 | 0.55 | Putative RND family drug transporter | |
| A1S_0538 | 0.06 | 0.66 | Putative RND family drug transporter | |
| A1S_0774 | 0.11 | 1.41 | Putative RND family drug transporter* | |
| A1S_0908 | 0.22 | 0.07 | RND family multidrug resistance secretion protein | |
| A1S_1241 | -0.73 | 0.91 | Putative RND family drug transporter | |
| A1S_1243 | -0.67 | 1.23 | Putative RND family drug transporter* | |
| A1S_1649 | -0.19 | 0.53 | Putative RND family drug transporter | |
| A1S_1769 | 0.75 | 0.28 | Putative RND family drug transporter | |
| A1S_1773 | 0.13 | 0.59 | RND family drug transporter | |
| A1S_1800 | -0.39 | 0.41 | Putative RND family drug transporter | |
| A1S_2304 | -0.06 | 0.47 | Putative RND family drug transporter | |
| A1S_2306 | 0.14 | -0.14 | Putative RND family drug transporter | |
| A1S_2618 | -0.31 | 0.89 | Putative RND family drug transporter | |
| A1S_2619 | 0.12 | 1.53 | Putative RND family drug transporter* | |
| A1S_2620 | 0.10 | 1.50 | Putative RND family drug transporter* | |
| A1S_2660 | -0.20 | 0.38 | Putative RND family drug transporter | |
| A1S_2736 | -0.06 | -0.14 | RND family drug transporter | |
| A1S_3092 | -0.10 | 0.84 | Putative RND type efflux pump involved in aminoglycoside resistance (adeT) | |
| A1S_3217 | 0.41 | 0.22 | RND divalent metal cation efflux transporter | |
| A1S_3445 | 0.24 | 0.27 | Putative RND family cation/multidrug efflux pump | |
| A1S_3446 | 0.04 | 1.28 | Putative RND family cation/multidrug efflux pump* | |
| A1S_3447 | -0.35 | 0.63 | Putative RND family drug transporter | |
| A1S_0237 | 0.01 | 0.61 | D-alanyl-D-alanine endopeptidase penicillin-binding protein 7 and penicillin-binding protein 8 | |
| A1S_1020 | 0.01 | -0.70 | Penicillin-binding protein 2 | |
| A1S_2435 | -0.06 | -0.90 | D-ala-D-ala-carboxypeptidase; penicillin-binding protein 5 (precursor) | |
| A1S_2479 | -0.18 | -0.36 | Putative D-ala-D-ala-carboxypeptidase penicillin-binding protein | |
| A1S_3196 | -0.20 | -0.07 | Putative penicillin binding protein (PonA) | |
| A1S_3197 | -0.32 | 0.30 | Putative penicillin binding protein (PonA) | |

**The differentially expressed genes with the threshold |log2(fold change)| > 1.*


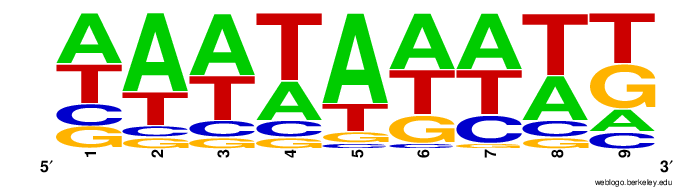


FIGURE S1. Consensus pattern of IS*Aba*1 insertion site sequences found in *A. baumannii* AYE genome as well as in *E. coli* J53 transconjugants following mating-out with *E. coli* RZ211 containing the recombinant plasmid pIS*Aba*1-TEM-1 (Mugnier et al., 2009). A total of 24 IS*Aba*1 insertion site sequences, including GAATAAACG, TTTTGTCTG, GAAAAATAC, CAAAAACAG, AAGCAGATT, TGTGATGAG, ACCGACAAG, AAAAATTTG, GAATATTCT, AAAAAGAAA, GATTAGCTC, TATTTTTCT, TAAAAACTT, TTTTGATGG, AGCTTAAAT, AATTTTCTT, AAATAGTTA, TATTTTTTC, CCATAAATT, CTAAAGAAA, CACCCAAA, ATGCAAAGG, ATATAAATT and TTTATTTTT, were identified and then analyzed by using Weblogo software (http://weblogo.berkeley.edu/logo.cgi). A consensus pattern WA(A/T)(A/T)A(A/T)(A/T)(A/T)W was proposed. W represents G, C, T, or A.

**Supplementary References**

De Gregorio, E., Del Franco, M., Martinucci, M., Roscetto, E., Zarrilli, R., and Di Nocera, P. P. (2015). Biofilm-associated proteins: news from *Acinetobacter*. BMC Genomics. 16: 933. doi: 10.1186/s12864-015-2136-6

Kuo, H. Y., Chang, K. C., Kuo, J. W., Yueh, H. W., and Liou M. L. (2012). Imipenem: a potent inducer of multidrug resistance in *Acinetobacter baumannii*. Int J Antimicrob Agents. 39, 33-38. doi: 10.1016/j.ijantimicag.2011.08.016

Mugnier, P. D., Poirel, L., and Nordmann, P. (2009). Functional analysis of insertion sequence IS*Aba*1, responsible for genomic plasticity of *Acinetobacter baumannii*. J Bacteriol. 191, 2414-2418. doi: 10.1128/JB.01258-08
